# Multimodal Building Damage Assessment Method Fusing Adaptive Attention Mechanism and State-Space Modeling

**DOI:** 10.3390/s26020638

**Published:** 2026-01-18

**Authors:** Rongping Zhu, Xiaoji Lan

**Affiliations:** 1 School of Civil and Surveying Engineering, Jiangxi University of Science and Technology, Ganzhou 341000, China; 6720230190@mail.jxust.edu.cn; 2 Technology Innovation Center for Land Spatial Ecological Protection and Restoration in Great Lakes Basin (JX), Ganzhou 341000, China

**Keywords:** building damage assessment, MultiModal Fusion, optical-SAR, SSM, Mamba, Siamese Network, adaptive attention, knowledge graph

## Abstract

Rapid and reliable building damage assessment (BDA) is crucial for post-disaster emergency response. However, existing methods face challenges such as complex background interference, the difficulty in jointly modeling local geometric details and global spatial dependencies, and adverse weather conditions. To address these issues, this paper proposes the Adaptive Difference State-Space Fusion Network (ADSFNet), capable of processing both optical and Synthetic Aperture Radar (SAR) data to alleviate weather-induced limitations. To achieve this, ADSFNet innovatively introduces the Adaptive Difference Attention Fusion (ADAF) module and the Hybrid Selective State-Space Convolution (HSSC) module. Specifically, ADAF integrates pre- and post-disaster features to guide the network to focus on building regions while suppressing background interference. Meanwhile, HSSC synergizes the local texture extraction of CNNs with the global modeling strength of Mamba, enabling the simultaneous capture of cross-building spatial relationships and fine-grained damage details. Experimental results on sub-meter high-resolution MultiModal (BRIGHT) and optical (xBD) datasets demonstrate that ADSFNet attains F1 scores of 71.36% and 73.98%, which are 1.29% and 0.6% higher than the state-of-the-art mainstream methods, respectively. Finally, we leverage the model outputs to construct a disaster-centric knowledge graph and integrate it with Large Language Models to develop an intelligent management system, providing a novel technical pathway for emergency decision-making.

## 1. Introduction

Following a natural disaster, building damage assessment is a critical means for quantifying the scope and severity of the disaster’s impact and plays a pivotal role in post-disaster emergency response, reconstruction planning, and urban safety management [1,2]. Remote sensing-based building damage assessment involves identifying structural or morphological changes in buildings by comparing multi-temporal, pre- and post-disaster imagery [3,4].

Traditional damage assessment methods primarily rely on manual surveys or ground inspections, which suffer from low efficiency, high operational risk, and strong subjectivity [5,6,7]. Consequently, these methods are inadequate for the stringent rapid decision-making requirements of the post-disaster “golden rescue period”. With the rapid advancement of high-resolution remote sensing and artificial intelligence, automated building damage identification based on remote sensing imagery has emerged as a key research direction in disaster emergency response [8,9]. Early automated detection methods typically employed machine learning techniques [10], such as Support Vector Machines (SVM) [11] and Random Forests (RF) [12]. These approaches generally relied on hand-crafted shallow features (e.g., spectral and texture features) to identify post-disaster changes [13]. However, due to limited feature representation, they are often inadequate for handling the complex, multi-scale, and non-linear change patterns found in real-world disaster scenarios [14].

In recent years, Deep Learning (DL) has demonstrated outstanding performance in remote sensing image analysis [15], with Convolutional Neural Networks (CNNs) widely applied to tasks such as object detection [16], semantic segmentation [17], and change detection [18,19,20]. Although deep learning offers significant advantages in feature extraction, early progress was constrained by the scarcity of large-scale annotated datasets. In 2019, Gupta et al. [21] released the xBD dataset, the first large-scale, high-resolution dataset featuring multiple disaster types, regional diversity, and paired pre- and post-disaster images. This dataset established a solid data foundation for the field and significantly promoted the development of subsequent algorithms.

Building on this foundation, researchers have proposed various innovative architectures to enhance feature representation capabilities. Addressing the limitations of feature extraction, Bai et al. [22] proposed an end-to-end model named PPM-SSNet, which achieves high-precision automated assessment by integrating dilated convolutional residual blocks, Squeeze-and-Excitation (SE) blocks, and a semi-Siamese network. Focusing on multi-scale feature fusion, Shen et al. [23] designed a multi-scale CNN named BDANet, creatively incorporating a cross-directional attention mechanism to effectively capture feature information in multiple directions. Furthermore, to improve global feature extraction efficiency, Seydi et al. [24] adopted a lightweight architecture based on a modified Coat-Net, utilizing self-attention mechanisms to efficiently process single post-disaster high-resolution imagery. Exploring the potential of Transformer architectures, Chen et al. [25] proposed a Siamese Transformer-based dual-task framework (DamFormer) for multi-temporal building damage assessment, demonstrating its effectiveness on the large-scale xBD dataset.

Meanwhile, related research has also focused on improving localization accuracy and fine-grained classification capabilities. Wu et al. [26] proposed an attention-based U-Net, leveraging image pairs to simultaneously achieve building localization and multi-level damage classification. Zheng et al. [27] developed a unified semantic change detection network, integrating deep localization and damage classification networks, and employing object-based post-processing to ensure semantic consistency. Addressing the class imbalance problem, Berezina and Liu [28] proposed a coupled CNN model combining U-Net for contour extraction and a ResNet-based classifier using focal loss, achieving high-precision assessment from high-resolution satellite imagery.

More recently, the research focus has gradually shifted towards handling complex, heterogeneous disaster scenarios. Wang et al. [29] addressed the lack of modeling for specific disaster patterns by proposing the U2DDS-Net, which integrates a Disaster Type Token (DT Token) and a Difference Swin Stage Module (DSSM) to achieve precise assessment across diverse scenarios. Wang et al. [30] further resolved the lack of prior position constraints by proposing the PCDASNet. This network integrates building extraction and classification through a cascaded framework and leverages adaptive differential attention to achieve interpretable, high-precision assessment.

Notably, integrating multi-source data has become a key trend to overcome the limitations of single sensors. Yu et al. [31] developed the MCDD model, utilizing multi-source information—including SAR indicators (ADI, DP), optical NDBI changes, and seismic PGA data—to perform intelligent assessment of earthquake damage. Kimijima et al. [32] proposed a gradual updating framework integrating Real-time Earthquake Building Damage Estimation (REBDE) with satellite-based ALOS-2 assessments, significantly enhancing timeliness. In terms of data resources, Rahnemoonfar et al. [33] released the RescueNet UAV dataset, providing detailed pixel-level annotations to assist rescue teams. More importantly, addressing the vulnerability of optical data to adverse weather, Chen et al. [34] released BRIGHT, the world’s first publicly available optical-SAR multimodal dataset. They proposed a framework decoupling localization and classification, utilizing Lovász loss [35] to optimize key decision classes, thereby achieving all-weather assessment.

However, existing building damage assessment methods still face two main challenges in moving towards all-weather, high-precision applications. On the one hand, at the architectural level, traditional Convolutional Neural Networks (e.g., BDANet and PPM-SSNet) are limited by their local receptive fields, making it difficult to capture the long-range spatial dependencies and chain-reaction damage patterns among building clusters. While Transformers (e.g., DamFormer) possess excellent global modeling capabilities, their quadratic computational complexity increases rapidly with image resolution, making them unsuitable for large-scale rapid response. Furthermore, although CNN + Transformer hybrids (e.g., U2DDS-Net) attempt to combine local and global features, they often struggle to effectively model fine-grained cross-building correlations in high-resolution scenarios due to the loss of spatial resolution in deep Transformer layers or the excessive computational overhead required for dense global attention. The emerging Mamba (State-Space Model) architecture excels at modeling long-range dependencies but risks disrupting the spatial continuity of local neighborhoods during the feature serialization process, leading to the loss of geometric details crucial for damage assessment.

On the other hand, although multimodal fusion is a promising trend, research remains in a nascent stage due to profound domain gaps between optical and SAR imagery. Fundamental differences in imaging geometry introduce SAR-specific distortions like layover and foreshortening, while high off-nadir angles in optical data cause building leaning and misregistration. Radiometric heterogeneity further complicates alignment: optical spectra are vulnerable to cloud occlusion, whereas SAR backscatter is often plagued by speckle noise. Consequently, methodologies for deep synergistic fusion remain extremely scarce. In summary, existing methods struggle to achieve robust, all-weather, and high-precision damage assessment, particularly in complex multimodal scenarios. To address these challenges, this paper proposes a Multimodal Adaptive Difference State-Space Fusion Network (ADSFNet). First, to enhance the stability of feature representation during the fusion stage, we design the Adaptive Difference Attention Fusion (ADAF) module. Utilizing building localization features as priors, this module combines a difference mechanism with adaptive attention to highlight critical regions while effectively suppressing background noise. Second, to overcome architectural limitations, we introduce the Hybrid Selective State-Space Convolution (HSSC) module. Adopting a parallel Mamba–CNN architecture, this module synergizes the local texture extraction of CNNs with the global modeling capabilities of Mamba, thereby achieving cross-building spatial correlation modeling and fine-grained damage recognition. A structured comparison between ADSFNet and representative state-of-the-art methods regarding architectures, modalities, and addressed limitations is provided in Table 1.

Furthermore, given that existing models mostly output pixel-level classification results lacking semantic understanding and structured knowledge association—making them difficult for emergency decision-makers to interpret directly—we explicitly address this gap. To extend the application value of our research, we construct a disaster information management and intelligent analysis framework based on a Knowledge Graph (KG) and Large Language Models (LLMs), providing a novel technical pathway for disaster understanding and emergency decision-making.

## 2. Research Methods

### 2.1. Overall Architecture of ADSFNet

**Figure 1:**
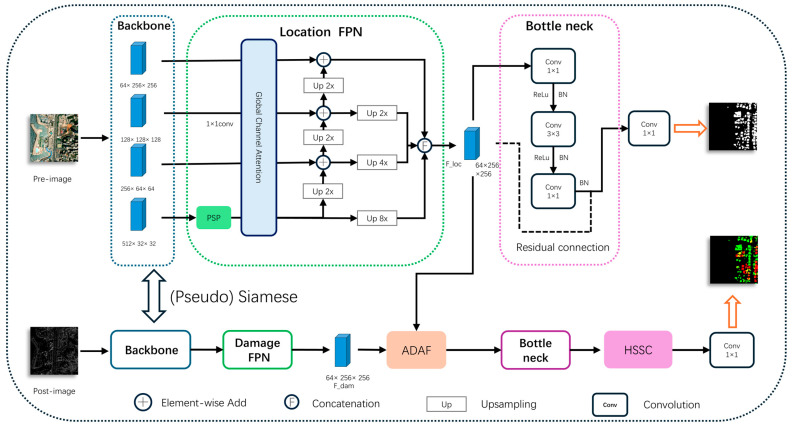
Overall framework of ADSFNet. Dotted boxes and arrows represent network modules and data flow, respectively.

The overall structure of ADSFNet is illustrated in Figure 1. The network employs a (Pseudo) Siamese encoder architecture based on ResNet34 (where the backbone operates in a Siamese mode for the optical xBD dataset and a Pseudo-Siamese mode for the multimodal BRIGHT dataset), consisting of two parallel task branches: the Building Localization Branch and the Damage Assessment Branch. These two branches are coupled via the Adaptive Difference Attention Fusion (ADAF) module, thereby forming an end-to-end joint prediction framework.

Formal Definition: Given a pre-disaster image $I_{pre}\in R^{H\times W\times 3}$ and a post-disaster image $I_{post}\in R^{H\times W\times 3},$ the model aims to output a building localization mask $M_{loc}\in R^{H\times W\times 1}$ and a multi-class damage assessment map $M_{cls}\in R^{H\times W\times C}$ (where C denotes the number of damage levels).

The specific workflow is as follows:

Feature Extraction and Pyramid Construction: $I_{\mathrm{pre}}$ and $I_{\mathrm{post}}$ are first fed into a dual-stream ResNet34 backbone to extract multi-scale features. Subsequently, two separate Feature Pyramid Networks (FPNs) perform top-down fusion on the multi-level features, generating localization features and initial damage features, respectively, which contain rich semantic and spatial details.

Building Localization Branch: This branch focuses on processing pre-disaster features. After being refined by a bottleneck module, the localization features are directly passed through a convolutional layer to generate the building localization mask $M_{loc}$. Simultaneously, these features serve as a structural prior and are forwarded to the damage branch to assist in assessment.

Damage Assessment Branch: This branch is based on post-disaster features. First, the Adaptive Difference Attention Fusion (ADAF) module is introduced as a key linkage between the two branches. It accepts localization features as structural priors and initial damage features from the current branch. ADAF utilizes an adaptive weighting mechanism to achieve deep cross-branch interaction and fusion of localization and damage information. The fused feature is refined by a bottleneck module before being input into the Hybrid Selective State-Space Convolution (HSSC) module. HSSC synergizes the global modeling capability of Mamba with the local feature extraction capability of CNNs, enhancing the structural understanding of contiguous building damage in complex scenarios. Finally, the enhanced feature is processed by a classifier to generate the final damage severity map $M_{loc}$.

### 2.2. Feature Extraction

The feature extraction module employs a (Pseudo) Siamese architecture based on ResNet34 to extract features from pre-disaster and post-disaster images, respectively. To accommodate the modal heterogeneity, referencing the baseline implementation of the BRIGHT dataset [34], we adopted a differentiated input strategy: while the pre-disaster branch processes standard three-channel optical images, the first convolutional layer of the post-disaster backbone is modified to accept single-channel SAR inputs directly. This design enables the model to effectively extract features from raw SAR intensity data while preserving its original radiometric characteristics. The encoder consists of one initial convolutional stem followed by four residual stages, progressively extracting textural details and semantic features from shallow to deep layers: shallow features capture building edges and morphology, whereas deeper features characterize overall structural and semantic attributes.

To enhance multi-scale feature representation and fusion, Feature Pyramid Networks (FPN) are introduced at the encoder output. The FPN integrates high-level semantic information with low-level spatial details through a top-down pathway and lateral connections. This process generates semantically rich and spatially enhanced feature maps, providing abundant multi-scale information for subsequent modules.

### 2.3. Adaptive Difference Attention Fusion Module

In building damage assessment tasks, models face two primary challenges. First, in general disaster scenarios (e.g., floods or earthquakes), buildings are often blurred or structurally incomplete due to occlusion or destruction, resulting in missed and false detections. Second, in multimodal remote sensing data, disparities in feature representation across different modalities often hinder effective information fusion.

To address these challenges, we design the Adaptive Difference Attention Fusion (ADAF) module. This module leverages pre-disaster localization features as a structural prior, explicitly captures temporal changes through a difference operation, and employs an adaptive weighting mechanism to dynamically adjust the contribution of features from both branches. This process highlights key change regions while effectively suppressing background interference.

**Figure 2:**
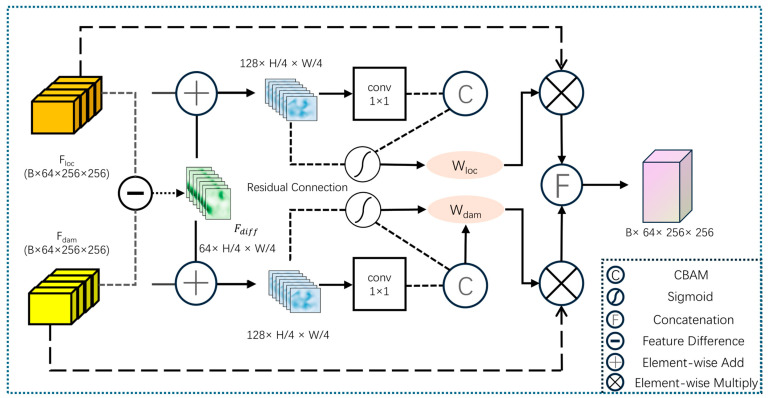
Structure of the ADAF module. The dotted box **encloses** the ADAF module operations, and arrows represent the data flow.

As illustrated in Figure 2, the ADAF module first performs a difference operation between the localization feature $F_{loc}$ and the damage feature $F_{dam}$ to explicitly capture the change information pre- and post-disaster:

(1) $$F_{diff}=F_{dam}-F_{loc}$$

Subsequently, the difference feature $F_{diff}$ is concatenated with the localization feature and the damage feature, respectively, along the channel dimension and then fed into a dual-branch convolutional structure for feature enhancement. Each branch incorporates a Convolutional Block Attention Module (CBAM), configured with a channel reduction ratio of 8 and a spatial kernel size of 7 × 7, combining channel and spatial attention to focus on salient change regions while retaining original semantic cues through residual connections.

The module further employs a Sigmoid activation function to generate adaptive weights, balancing the contributions of the two features:

(2) $$W_{loc}=\sigma \left(\mathrm{Conv}\left(F_{loc}^{\prime }\right)\right)$$

(3) $$W_{dam}=\sigma \left(\mathrm{Conv}\left(F_{dam}^{\prime }\right)\right)$$

where ${F\prime }_{loc}$ and ${F\prime }_{dam}$ denote the features enhanced by CBAM and residual connections, σ represents the Sigmoid activation function, and Conv denotes a convolution operation.

Finally, the original input features are modulated by their corresponding adaptive weights via element-wise multiplication, concatenated along the channel dimension, and processed by a fusion convolution to produce the final output:

(4) $$F_{out}={\mathrm{Conv}}_{\mathrm{cat}}\left(\left[F_{loc}⊙W_{loc},|\;F_{dam}⊙W_{dam}\right]\right)$$

where $cat\left(\cdot \right)$ represents channel-wise concatenation, and ⊙ denotes element-wise multiplication.

Through this design, the ADAF module significantly enhances the representation of salient change regions while suppressing background noise, thereby improving the model’s sensitivity and robustness in complex post-disaster scenarios. The fused result is then processed by a bottleneck module for semantic refinement. Utilizing a “1 × 1 − 3 × 3 − 1 × 1” convolutional structure, this bottleneck compresses and reconstructs the features, enhancing non-linear representation capacity while maintaining computational efficiency, ultimately providing high-quality feature maps for the subsequent HSSC module.

### 2.4. Hybrid Selective State-Space Convolution Module

In disaster scenarios, building clusters often exhibit complex spatial dependencies and cascading damage patterns. Single CNN architectures, constrained by local receptive fields, struggle to capture long-range dependencies. Conversely, pure SSM architectures, while offering strong global modeling, risk compromising local texture and boundary information during the feature flattening process. To resolve this architectural trade-off, we propose the Hybrid Selective State-Space Convolution (HSSC) module. Instead of traditional sequential stacking, this module establishes a parallel complementary mechanism. It aims to explicitly decouple global semantic modeling from local geometric extraction, processing them via distinct heterogeneous branches, and finally enabling deep feature interaction through channel reorganization.

**Figure 3:**
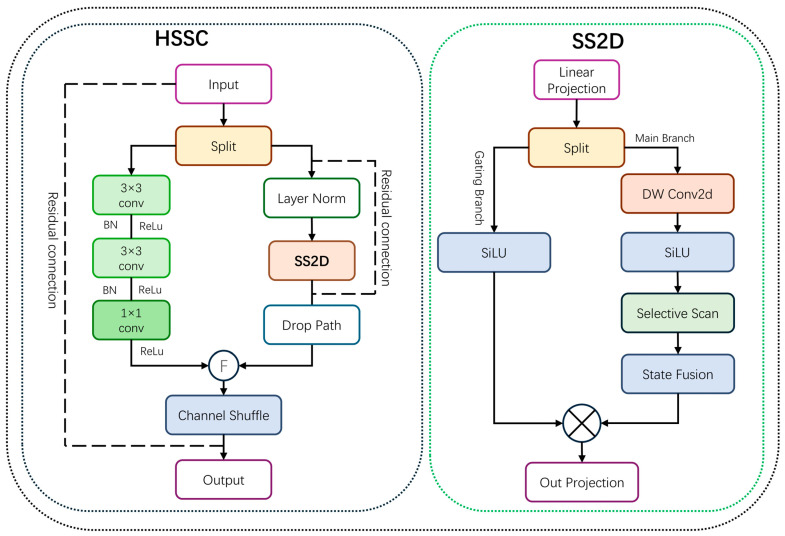
Structure of the HSSC module. Dotted boxes represent the internal architecture of the HSSC module and the detailed expansion of its SS2D component; arrows indicate the direction of data flow.

As illustrated in Figure 3, the HSSC module employs a ‘Parallel-Concatenate-Shuffle’ strategy. The input bottleneck feature $F_{in}$ is split along the channel dimension into two components with a balanced split ratio of 1:1:

(5) $$\left[X_{ss2d},X_{cnn}\right]=\mathrm{Split}\left(F_{in}\right)$$

State-Space Branch: This branch processes the first component,$X_{ss2d}$, using the SS2D mechanism of Vision Mamba. Configured with a state dimension of 16, a local convolution kernel size of 3, and an expansion factor of 2, it traverses spatial tokens in four directions efficiently, capturing long-range context across buildings with linear computational complexity to model cascading damage patterns.

Local Convolution Branch: Simultaneously, the second component, $X_{cnn}$, is handled by a local convolutional network composed of consecutive layers with a kernel size of 3 × 3. Leveraging the inductive bias of convolutional layers, this branch focuses on extracting high-frequency building edges and textural details, explicitly compensating for the limitations of the SSM branch in local feature characterization.

To break the information barrier between the parallel branches, a Channel Shuffle operation is introduced after concatenating the outputs $Y_{global}$ and $Y_{local}$. This step facilitates information flow across channel groups, enabling the deep fusion of global and local information. Finally, a residual connection with the input feature is added to produce the module output:

(6) $$F_{out}=\mathrm{Shuffle}\left(cat\left(Y_{global},Y_{local}\right)\right)+F_{in}$$

where $\mathrm{Split}\left(\cdot \right)$ denotes splitting along the channel dimension, $cat\left(\cdot \right)$ denotes channel-wise concatenation, and $Shuffle\left(\cdot \right)$ represents the Channel Shuffle operation.

By jointly modeling global scanning and local convolution, HSSC achieves multi-scale context fusion and cross-building dependency modeling. This enables precise detection of heavily damaged structures, even in regions with cascading failures. Furthermore, it substantially improves both the discriminative power and stability of the model in complex post-disaster scenarios.

## 3. Experiments and Results Analysis

### 3.1. Datasets

To validate the effectiveness of the proposed method, the BRIGHT dataset and the xBD dataset were selected as experimental benchmarks. Both datasets contain paired pre- and post-disaster high-resolution remote sensing imagery but differ significantly in data modality, disaster types, and annotation schemes. These differences allow for a comprehensive evaluation of the model’s generalization and robustness across multimodal and multi-hazard scenarios.

#### 3.1.1. BRIGHT Dataset

The BRIGHT dataset is a global multimodal remote sensing benchmark specifically constructed for building damage assessment, covering 4246 pairs of high-resolution images from 14 different disaster events worldwide. Each sample pair consists of a pre-disaster optical image and a post-disaster Synthetic Aperture Radar (SAR) image, with a spatial resolution of approximately 0.3–1.0 m and image dimensions of 1024 × 1024 pixels.

This dataset exhibits high diversity in disaster types, geographic regions, and scene characteristics. Furthermore, there are significant imaging differences between the optical and SAR modalities, and the damage categories—classified into Intact, Damaged, and Destroyed—are highly imbalanced. These characteristics pose significant challenges for achieving high-precision assessment under multimodal conditions. To ensure the strict alignment required for pixel-level fusion, we excluded the Ukraine, Myanmar, and Mexico subsets. Unlike standardized data, these regions necessitate a complex manual pipeline (historical imagery acquisition via Google Earth Pro and manual georeferencing, as detailed in the official documentation), which introduces uncontrollable geometric distortions. Such interventions inevitably lead to alignment discrepancies between modalities, causing severe feature mismatching that compromises experimental rigor.

#### 3.1.2. xBD Dataset

The xBD dataset is the first large-scale public remote sensing dataset for building damage assessment, released by the xView2 challenge and provided by the Maxar Open Data Program. This dataset covers 19 major natural disaster events worldwide, including six typical disaster types such as tsunamis, typhoons, floods, earthquakes, volcanoes, and fires. It spans a total area of more than 45,000 km^2^ and includes imagery from 15 countries.

xBD contains a total of 22,068 high-resolution satellite images of 1024 × 1024 pixels. Each sample consists of pre-disaster and post-disaster optical images. Each building instance is annotated with a polygon and a unique identifier and is labeled in the post-disaster image with one of four damage levels: No Damage, Minor Damage, Major Damage, and Destroyed.

The building classes in this dataset also exhibit significant imbalance: background pixels constitute approximately 97% of the total pixels, and among building pixels, the number of No Damage samples greatly outnumbers the other three classes. Such severe class imbalance poses significant challenges to model training, necessitating dedicated strategies for class balancing in both architecture design and loss function formulation.

### 3.2. Experimental Results and Analysis

#### 3.2.1. Implementation Details

All experiments in this study were conducted using the PyTorch (version 2.7.1) deep learning framework on a workstation equipped with an NVIDIA RTX 4090 GPU (Nvidia, Santa Clara, CA, USA) and CUDA 11.8. The input images were maintained at their original resolution of 1024 × 1024 pixels. To enhance model generalization and prevent overfitting, a diverse set of data augmentation strategies was applied during training, including random horizontal and vertical flipping, random rotation, random cropping, multi-scale scaling, color jittering (brightness, contrast, saturation), and adding Gaussian noise.

Regarding data modality, distinct preprocessing schemes were adopted. For the xBD dataset, both pre- and post-disaster optical images were normalized using ImageNet statistics and directly fed into the network. For the BRIGHT dataset, optical images utilized standard ImageNet normalization, whereas the post-disaster SAR images were processed following the standard protocols outlined by Chen et al. [34]. To accommodate this modality, the first convolutional layer of the post-disaster backbone was structurally modified to accept single-channel input directly, enabling the network to extract features from raw SAR intensity data.

The network was optimized using the AdamW optimizer with an initial learning rate of 1 × 10^−3^. A polynomial decay strategy (power = 0.9) was employed for dynamic learning rate scheduling. Considering the difference in dataset scale, the maximum number of training iterations was set to 120,000 for xBD and 70,000 for BRIGHT.

#### 3.2.2. Loss Function

Given the multi-task nature of our framework, which simultaneously performs building localization and damage assessment, we formulate a joint optimization objective. The total loss $L_{total}$ is defined as the weighted sum of the localization loss $L_{loc}$ and the damage classification loss $L_{cls}$:

(7) $$L_{total}=L_{loc}+\lambda L_{cls}$$

where λ is a hyperparameter used to balance the two tasks. Based on empirical experiments, we set λ = 3.0 to prioritize the optimization of the more challenging damage assessment task.

Specifically, for the building localization branch, we employ the standard Cross-Entropy Loss to supervise the binary segmentation of building footprints. For the damage assessment branch, we employ a hybrid loss combining Weighted Focal Loss [36] and Lovász-Softmax Loss to address class imbalance:

(8) $$L_{cls}=L_{Focal}+0.75L_{Lovasz}$$

We tailored the class weights in Focal Loss to the specific definitions of each dataset:

For BRIGHT (4 classes: Background, Intact, Damaged, Destroyed), we set weights to [1, 1, 4, 2]. The highest weight (4.0) is assigned to the ‘Damaged’ class, which is the most challenging to distinguish.

For xBD (5 classes: Background, No damage, Minor, Major, Destroyed), we set weights to [1, 1, 4, 2, 2]. Consistent with BRIGHT, the Minor-damage class is weighted 4.0 due to its high detection difficulty, while Major-damage and Destroyed are both weighted 2.0 to emphasize severe damage types.

#### 3.2.3. Evaluation Metrics

To comprehensively evaluate the model’s performance on both building localization and damage classification tasks, this study adopts the standard evaluation metric system from the xView2 Challenge, with the F1-score serving as the core metric. The F1-score combines Precision and Recall, defined as follows:

(9) $$F1=\frac{2\times \mathrm{Precision}\times \mathrm{Recall}}{\mathrm{Precision}+\mathrm{Recall}}$$

(10) $$\mathrm{Precision}=\frac{TP}{TP+FP}$$

(11) $$\mathrm{Recall}=\frac{TP}{TP+FN}$$

where TP, FP, and FN denote the number of True Positives, False Positives, and False Negatives, respectively.

The evaluation system assesses model performance at two levels:

Building localization accuracy $F1_{loc}$: measuring the accuracy of building extraction.

Building damage classification accuracy $F1_{cls}$: evaluating the classification performance for different building damage levels.

The comprehensive Score is computed as a weighted sum of these two metrics:

(12) $$\mathrm{Score}=0.3\times F1_{loc}+0.7\times F1_{cls}$$

where $F1_{cls}$ denotes the harmonic mean of the F1-scores over all damage categories:

(13) $$F1_{cls}=\frac{N}{\sum_{i=1}^{N}\frac{1}{F1_{i}}}$$

Here, N denotes the number of damage categories, and $F1_{i}$ is the F1-score of the i-th class.

This weighted metric appropriately balances localization accuracy and multi-class classification performance, providing a holistic indicator of the model’s overall capability in complex disaster assessment tasks.

#### 3.2.4. Results on the BRIGHT Dataset

To further validate the model’s robustness and generalization capabilities under multimodal conditions, experiments are conducted on the BRIGHT dataset. This dataset integrates pre-disaster optical imagery with post-disaster SAR imagery, characterized by modal heterogeneity and speckle noise, thereby imposing higher demands on the model’s feature alignment and cross-modal fusion capabilities.

To quantitatively evaluate the performance of various methods, Table 2 presents the building damage assessment results on the BRIGHT dataset. The results show that ADSFNet achieves the best overall performance, with an Overall Score of 71.36%, surpassing the second-best model by 1.29%. Although DamFormer exhibits competitive performance in building localization and intact building recognition, these gains are accompanied by high computational complexity and parameter burden. In comparison, ADSFNet achieves a better trade-off between efficiency and accuracy, demonstrating a clear advantage in the challenging Damaged category.

In contrast, conventional networks (e.g., DeepLabv3+, U-Net) are primarily designed for single-modal optical imagery. When processing SAR imagery, these models are highly susceptible to SAR speckle noise, resulting in blurred building boundaries and inaccurate damage discrimination. ADSFNet, however, effectively leverages the complementarity of multimodal features through its structural design:

On the one hand, the ADAF module utilizes building localization features to impose strong spatial constraints. This plays a critical role in filtering out SAR speckle noise and aligning building boundaries, thereby enhancing the accuracy of cross-modal fusion.

On the other hand, the HSSC module incorporates long-range context to compensate for the deficiency of local features in capturing global dependencies, ensuring that the model generates structurally complete and semantically continuous damage predictions, especially in regions with contiguous building damage.

**Figure 4:**
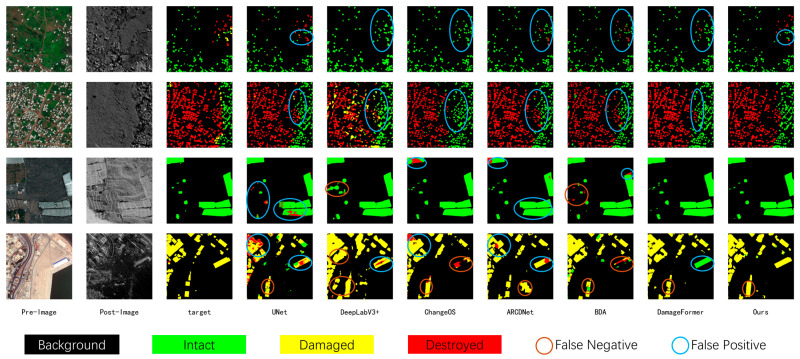
Visual Results on BRIGHT dataset.

As shown in the visualization results (Figure 4), ADSFNet exhibits superior prediction consistency and internal integrity in multimodal imagery. When identifying large-scale buildings or contiguous damaged regions (as indicated by the marked regions), the predictions of comparison models (e.g., U-Net and DeepLabV3+) often exhibit fragmented characteristics. This means that multiple incorrect categories are mixed within a single building or disaster zone, resulting in a “speckled” distribution of predictions. In contrast, ADSFNet effectively perceives contiguous building clusters as a cohesive whole. Its predicted damaged regions appear uniform in color with clear boundaries, effectively eliminating prediction holes and noisy clutter within the building structures. This precise grasp of structural integrity directly reflects the long-range modeling advantage of the HSSC module—it enables the model to look beyond local pixels and leverage broader contextual information to constrain prediction consistency, thereby maintaining accurate and robust damage assessment even amidst the heterogeneous interference of multimodal data.

#### 3.2.5. Results on the xBD Dataset

Table 3 presents the quantitative comparison results on the xBD dataset, Comparing ADSFNet with classic CNN architectures such as U-Net [37], DeepLabv3+ [38], ChangeOS [27], BDANet [23], and the Transformer-based DamFormer [25]. As shown in the table, ADSFNet demonstrates superior overall performance, achieving an Overall Score of 73.98% and a building localization accuracy (F1_loc) of 87.67%. These results indicate that the network maintains superior boundary segmentation quality and structural integrity, even under single-modal optical conditions.

Regarding damage classification, the $F1_{cls}$ score improved to 68.12%. A significant performance leap was observed, particularly in the most challenging ‘Minor Damage’ category, which is typically difficult to recognize due to its subtle visual cues. This improvement is primarily attributed to the localization-guided feature refinement mechanism. Unlike traditional full-image feature extraction, this mechanism utilizes localization information to impose spatial constraints on damage features, effectively filtering out background noise interference in non-building areas. This significant enhancement in the signal-to-noise ratio (SNR) enables the model to extract easily overlooked minor damage features from complex backgrounds, thereby preventing weak signals from being submerged by environmental noise.

**Figure 5:**
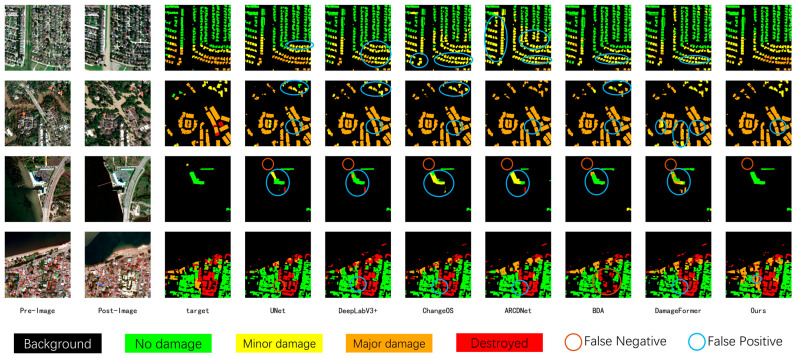
Visual Results on xBD dataset.

As illustrated in the visualization results (Figure 5), ADSFNet demonstrates distinct advantages in the precision of building localization and the sensitivity of damage recognition. In non-building areas, comparison models are prone to noticeable false positives, incorrectly predicting background textures as buildings. In contrast, ADSFNet effectively eliminates these false detections by leveraging explicit localization priors, ensuring that only actual building targets are extracted.

Regarding damage classification, other models often exhibit insensitivity to disaster features, frequently misclassifying actual damaged regions as “No Damage” (green). Conversely, ADSFNet, aided by its feature refinement mechanism, effectively identifies these overlooked damage features and accurately reflects the actual damage conditions, while avoiding the fragmented and inconsistent predictions commonly seen in other models. This fully validates the model’s effectiveness in utilizing localization priors to filter out interference and precisely capturing weak damage signals.

### 3.3. Ablation Experiments

To validate the efficacy and synergistic effects of the proposed key modules, ablation experiments are conducted on the xBD dataset. The rationale for selecting the xBD dataset is twofold: First, as one of the largest benchmarks with the richest disaster types and highest scene diversity, performance metrics validated on xBD possess broad statistical representativeness and generalization significance. Second, as a single-modal optical dataset, xBD provides an ideal controlled environment. This allows for the independent evaluation of each module’s contribution without the interference of multimodal heterogeneity, thereby enabling a clearer analysis of the performance gains and synergistic effects. The experiments are conducted on a dual-branch baseline network, with ADAF and HSSC incorporated separately to evaluate their individual and combined impacts.

The quantitative results are presented in Table 4. Incorporating ADAF and HSSC into the baseline network yields distinct performance improvements.

With the sole inclusion of ADAF, the model outperforms the baseline in Minor Damage ($F1_{min}$) and Major Damage ($F1_{maj}$), with $F1_{maj}$ rising to 69.54%. This indicates that ADAF, by incorporating localization priors, successfully redirects the model’s attention focus from complex background regions to the building structures.

With the sole inclusion of HSSC, the Destroyed ($F1_{des}$) category achieves the highest performance among single-module configurations (75.87%). The HSSC utilizes its long-range modeling mechanism to capture contextual information surrounding building clusters (e.g., the propagation trends of collapsed debris). This deep exploitation of spatial neighborhood relationships enables the model to infer damaged buildings that are difficult to identify based on local cues alone, significantly enhancing boundary delineation in regions with contiguous collapse.

When both modules are integrated, their combined strengths are fully leveraged. The Overall Score improves to 73.98%, and notably, the F1-score for the most challenging Minor Damage category achieves a significant breakthrough, reaching 52.19%. These results quantitatively confirm that ADAF and HSSC demonstrate strong functional complementarity, collectively driving the model to achieve optimal performance in multi-class damage assessment tasks.

**Figure 6:**
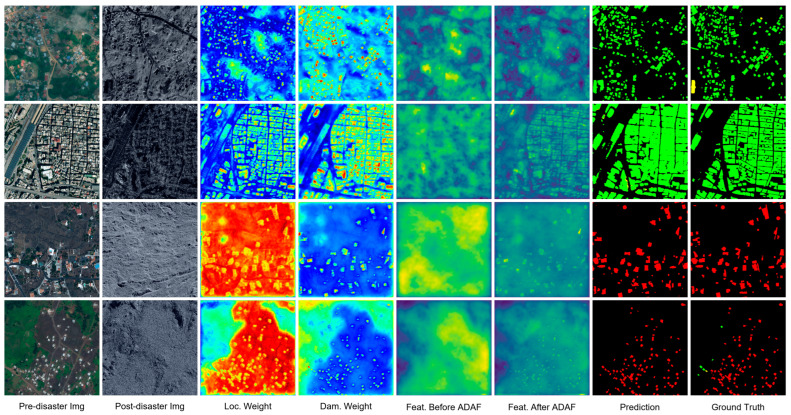
Visualization of the ADAF internal mechanism.

While the quantitative results on the xBD dataset confirm the performance gains in optical scenarios, to strictly validate the ADAF module’s robustness in handling SAR speckle noise (a key motivation for its design), we further visualize its internal interpretability mechanism using multimodal data in Figure 6. We selected four representative disaster scenarios—Bata-explosion, Beirut-explosion, Congo-volcano, and La Palma-volcano—for this detailed analysis.

Feature Refinement: Comparing the feature maps, the features before ADAF (Feat. Before ADAF) exhibit diffuse and continuous activations covering almost the entire image. This indicates that without spatial guidance, the network observes the scene indiscriminately, failing to distinguish building locations from background clutter. In sharp contrast, the features after ADAF (Feat. After ADAF) show clear building boundaries with the background effectively suppressed, proving that the module successfully disentangles target features from noise.

Adaptive Weight Analysis: The visualization reveals how ADAF dynamically balances the contributions of pre- and post-disaster features based on image quality:

In high-quality SAR scenarios (e.g., Bata/Beirut): The module assigns comparable weights to both branches. This balanced assignment allows the model to simultaneously attend to the structural priors (from pre-disaster weight, *Loc_w_*) and the damage evidence (from post-disaster weight, *Dam_w_*), effectively balancing their contributions to generate accurate damage representations.

In low-quality SAR scenarios (e.g., Congo/La Palma): When the imagery is heavily corrupted by volcanic ash or complex noise, the model adaptively shifts its reliance towards the Pre-disaster Weight (Locw). As shown in the visualization, Locw serves as a dominant spatial anchor (high red/yellow response), essentially vetoing the unreliable noise in the post-disaster branch.

This mechanism confirms that ADAF acts as an intelligent regulator, ensuring that robust structural priors compensate for environmental interference when necessary.

## 4. Discussion and Application Extensions

Although the proposed ADSFNet has significantly improved the accuracy of damage assessment, pixel-level damage distribution maps alone often fail to meet the deeper informational needs of emergency decision-making. Existing studies predominantly focus on visual-level feature recognition, lacking effective means for the structured organization and semantic association of assessment results. Consequently, high-value remote sensing interpretation results cannot be rapidly translated into actionable intelligence to guide rescue operations. To address this limitation, building upon the proposed model, this study further explores the application extensions of our findings in disaster information management and intelligent decision-making.

**Figure 7:**
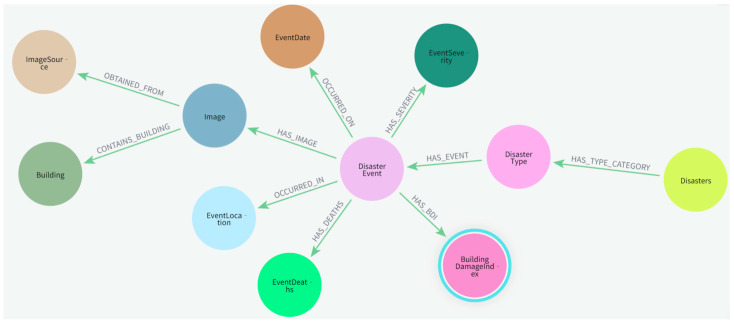
The ontology schema of the disaster knowledge graph.

To achieve semantic management and multi-dimensional association analysis of the model’s outputs, we constructed a disaster-centric Knowledge Graph (KG), with its data schema visualized in Figure 7, aiming to realize the structured storage, associative organization, and semantic expression of disaster information. The KG takes disaster events, building entities, damage indicators, and geospatial information as core nodes, constructing a multi-level semantic association network through attributes such as damage levels, timestamps, and disaster types. This architecture not only stores the prediction results of ADSFNet but also enables linked querying and knowledge reasoning across three dimensions: “macro-disaster events”, “meso-regional distribution”, and “micro-individual buildings”. Specifically, building nodes record spatial footprints and damage levels, image nodes associate geospatial coordinates, and disaster event nodes integrate metadata such as type, location, time, and intensity. This design forms an extensible and interpretable disaster knowledge system, providing solid data support for post-disaster damage statistics, regional assessment, and reconstruction planning.

**Figure 8:**
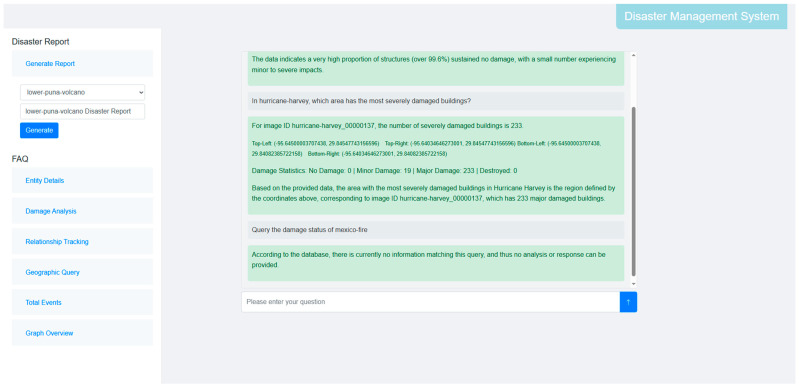
Prototype System for Disaster Management.

Based on this foundation, we developed a prototype system for disaster knowledge question-answering (QA) and report generation using the LangChain framework (as shown in Figure 8). This system innovatively integrates the structured data of the KG with the semantic understanding capabilities of Large Language Models (LLMs). Specifically, the system utilizes the LLM to parse natural language queries into structured Cypher queries, enabling precise information retrieval from the graph database. Using the KG as the factual basis and an LLM as the reasoning core, the system can accurately parse user queries and retrieve structured results, and perform multi-dimensional analysis based on the returned data. Crucially, given that the inherent “hallucination” (factual fabrication) tendencies of generative models pose unacceptable risks in safety-critical disaster management, our design imposes a strict reliance on the KG as the immutable ground truth. This ensures that all responses are rigorously anchored to verified graph data rather than generated from statistical probability. As validated in Figure 8, this factual anchoring allows the system to accurately extract statistics for valid queries (e.g., “Hurricane Harvey”) while explicitly activating a safety protocol to refuse out-of-domain inputs (e.g., “Mexico-fire”), thereby eliminating the risk of misleading intelligence. Furthermore, the prototype system can automatically generate disaster briefings based on the retrieved structured information, providing intelligent support for emergency response and decision-making.

This research marks a technological transition from “visual perception of imagery” to “cognitive reasoning of disaster knowledge,” demonstrating the profound value of deep learning outcomes in the organization and intelligent utilization of disaster information. By transforming remote sensing recognition results into queryable and reasoning-capable knowledge structures, this paper provides a novel technical pathway for multi-source information fusion and intelligent management in disaster scenarios.

## 5. Limitations and Future Work

Despite the robust performance demonstrated by ADSFNet, limitations persist in complex multimodal scenarios. Due to the inherent differences in physical imaging mechanisms between SAR and optical imagery, achieving precise feature alignment and semantic consistency across varying data distributions remains a challenge.

**Figure 9:**
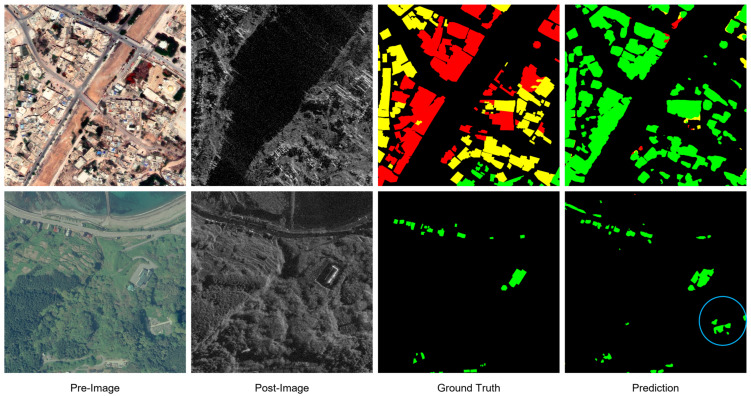
Visualization of typical failure cases.

As visualized in Figure 9, these challenges manifest across several key dimensions. In the flood scenario (Top row), where a significant portion of the central area is submerged, the specular reflection of SAR causes signal voids. This severe physical limitation leads to unavoidable false positives, indicating that standard SAR-based damage assessment faces applicability challenges in specific hydrological disasters. Conversely, in the earthquake scenario (Bottom row), discrepancies arise regarding target positioning. The model detected unannotated targets that exhibit high visual similarity to buildings in the pre-disaster optical imagery. While these targets possess distinct structural features, their misclassification indicates that the current feature representation lacks sufficient discrimination to separate true buildings from similar man-made objects. This suggests that further refining the robustness of the building extraction branch is necessary to effectively filter out such ambiguous distractors.

Furthermore, as highlighted by Ponzo et al. [39] and the ReLUIS technical guidelines [40], environmental factors such as air temperature variations can introduce significant interference in satellite-based monitoring for certain infrastructure types. Thermal loads on slender structures (e.g., long-span roofs, bridges, and viaducts) can induce deformations exceeding one-quarter of the radar wavelength, potentially leading to the loss or degradation of retrieved interferometric information. Distinguishing these physiological deformations from actual disaster-related damage is crucial for improving the interpretative value of multi-modal assessment.

Future research will focus on two key aspects:

Model Architecture: We plan to incorporate cross-modal generative models and domain adaptation mechanisms. This aims to bridge the distributional gap between heterogeneous modalities, thereby enhancing generalization performance in complex or few-shot scenarios.

Knowledge Integration: We will explore the construction and dynamic evolution of Spatiotemporal Knowledge Graphs. The goal is to establish an adaptive closed-loop system integrating perception, reasoning, and decision-making, enabling a transition from single-event assessment to full-cycle dynamic disaster management.

## 6. Conclusions

Addressing the limitations of existing building damage assessment methods—such as widespread reliance on single-modal imagery, insufficient accuracy in complex disaster scenarios, and missed detections caused by blurred or structurally incomplete post-disaster buildings—this paper proposes the Adaptive Difference State-Space Fusion Network (ADSFNet). Through the synergistic design of the Adaptive Difference Attention Fusion (ADAF) module and the Hybrid Selective State-Space Convolution (HSSC) module, the model achieves full utilization of building localization priors and joint modeling of global-local features. These designs collectively establish a unified framework that integrates spatial priors, cross-modal difference reasoning, and global–local feature fusion.

Experimental results demonstrate that ADSFNet achieves superior performance on both the xBD and BRIGHT representative datasets, attaining Overall Scores of 73.98% and 71.36%, respectively. These results significantly outperform existing mainstream methods (achieving a 1.29% improvement over the second-best model on the BRIGHT dataset). The consistent performance gains across these heterogeneous datasets not only validate the all-weather robustness of the proposed method in optical–SAR cross-modal scenarios but also demonstrate its strong generalization capability in handling challenging disaster data. At the same time, it is important to account for the impact of fundamental sensor-specific imaging mechanisms on the model’s performance. Specifically, the model’s efficacy is still inevitably influenced by the inherent physical constraints of SAR imaging, such as signal loss caused by specular reflection on water surfaces in specific flood scenarios, or thermal-induced structural noise in temperature-sensitive infrastructures. Acknowledging these boundary conditions provides a more objective and comprehensive evaluation of the framework’s high-reliability performance.

Furthermore, this study integrates model outputs with geographic information to construct a Disaster Knowledge Graph and develops a Knowledge QA and Report Generation System based on the LangChain framework and Large Language Models. This system not only demonstrates the practical potential of deep learning models in disaster information management but also provides a scalable and extensible technical framework, applicable to diverse disaster types and multi-source geospatial applications in future emergency response.

## Figures and Tables

**Table 1:** Comparison with representative methods.

Method	Modality	Core Mechanism	Addressed Limitations
ChangeOS [27]	Optical	Deep Object-Based Semantic Change Detection	Geometric Inconsistency: Risks losing details and complementarity.
BDANet [23]	Optical	Two-stage CNN + Cross-Directional Attention	Local Constraint: Fails to capture global spatial dependencies.
DamFormer [25]	Optical	Siamese Transformer Architecture	Computational Complexity: Inefficient for high-res rapid response.
PCDASNet [30]	Optical	Cascaded framework with Position-Constrained DAM	Single-modal Constraint: Ineffective under clouds, smoke, or night.
U2DDS-Net [29]	Optical	U2Net + Swin Transformer with Disaster-type Tokens	Heavy Architecture: 75 M parameters hinder real-time deployment.
ADSFNet(Ours)	Opt + SAR	Mamba-CNN Hybrid + Adaptive Difference Fusion	Proposed: Global-local synergy with all-weather robustness.

**Table 2:** Quantitative comparison results on the BRIGHT dataset.

Method	Score (%)	F1*_loc_* (%)	F1*_cls_* (%)	F1*_int_* (%)	F1*_dam_* (%)	F1*_d__es_* (%)
ARCDNet	66.77	88.59	57.41	88.50	40.87	60.66
Deeplabv3+	68.90	86.60	61.55	91.87	45.33	63.31
U-net	63.13	89.19	51.96	89.52	31.51	67.41
DamFormer	70.07	89.81	61.60	90.29	43.62	68.05
BDA	61.36	88.8	49.61	84.50	31.21	60.24
ChangeOS	61.72	84.92	51.77	83.65	34.11	59.96
Ours	71.36	88.87	63.86	89.72	46.80	69.16

**Table 3:** Quantitative comparison results on the xBD dataset.

Method	Score (%)	F1*_loc_* (%)	F1*_cls_* (%)	F1_no_ (%)	F1_min_ (%)	F1_maj_ (%)	F1_des_ (%)
ARCDnet	73.30	86.82	67.51	83.92	51.24	69.29	74.72
Deeplabv3+	71.97	87.02	65.52	83.98	47.64	68.21	74.12
U-net	71.49	87.13	64.78	84.15	47.04	66.04	74.30
DamFormer	70.50	85.45	64.10	83.98	46.75	66.63	70.93
BDA	73.38	87.42	67.37	84.65	50.53	68.90	75.42
ChangeOS	72.98	86.88	67.03	84.44	50.37	68.59	74.62
Ours	73.98	87.67	68.12	84.97	52.19	68.87	75.34

**Table 4:** Ablation study on the xBD dataset. Note:√ indicates that the corresponding module is incorporated into the network.

ADAF	HSSC	Score (%)	F1*_loc_* (%)	F1*_cls_* (%)	F1_no_ (%)	F1_min_ (%)	F1_maj_ (%)	F1_des_ (%)
		72.40	86.45	66.39	84.09	49.25	68.03	74.86
√		73.31	87.29	67.32	84.55	50.11	69.54	75.44
	√	73.27	87.72	67.08	84.92	49.31	69.46	75.87
√	√	73.98	87.67	68.12	84.97	52.19	68.87	75.34

## Data Availability

Publicly available datasets were analyzed in this study. The xBD dataset can be found at https://xview2.org/dataset (accessed on 29 December 2025). The BRIGHT dataset is available in the Zenodo repository at https://zenodo.org/records/15385983 (accessed on 1 September 2025).

## Abbreviations

SSM	State-Space Model
CNN	Convolutional Neural Network
CBAM	Convolutional Block Attention Module
SAR	Synthetic Aperture Radar
FPN	Feature Pyramid Network
LLM	Large Language Model
KG	Knowledge Graph
ADSFNet	Adaptive Difference State-Space Fusion Network
ADAF	Adaptive Difference Attention Fusion
HSSC	Hybrid Selective State-Space Convolution
